# MosaicLev: modified Levenshtein distance for mobile element-aware genome comparison

**DOI:** 10.1093/bioadv/vbag261

**Published:** 2026-09-07

**Authors:** Harry Stoltz, Thomas E Kuhlman

**Affiliations:** Department of Physics and Astronomy, University of California, Riverside, CA 92521, United States; Courant Institute of Mathematical Sciences, New York University, New York, NY 10012, United States; Department of Physics and Astronomy, University of California, Riverside, CA 92521, United States

## Abstract

**Motivation:**

Genomes diverge, in part due to the activity of mobile elements, including elements that excise and reinsert (cut–paste) or propagate via an RNA intermediate (copy–paste). Standard sequence comparison methods are not motif-aware, penalizing mobile element insertions based on length rather than recognizing them as single biological events, while alignment-free methods still fail to adequately describe a known mobile-element sequence as a unified change.

**Results:**

We introduce a modified Levenshtein distance (mlev) that discounts specified block insertions via a tunable parameter (m ∈ [0,1]): at m=0 it recovers ordinary Levenshtein distance and at m=1 a whole chunk acts like a single edit. On 67 Cluster G1 mycobacteriophage genomes (a viral group with MPME1 and MPME2), MPME1 targeting yielded ∼53% discount for the 31-genome MPME1-score group and ∼9% for the 11-genome MPME2-score group. MPME2 targeting reversed this pattern, with 18 phages low on both scores. For PV92, a 346-bp Yb8-containing insertion received 99.71% forward reduction at m=1 and none in reverse. Together, these results demonstrate that MosaicLev can quantify the contribution of known mobile elements to sequence differences across distinct genomic settings.

**Availability and implementation:**

Python implementation with Numba JIT compilation freely available at https://doi.org/10.5281/zenodo.18452982

## 1 Introduction

In this paper, we will provide a mathematical exploration into mobile element-aware sequence comparison. DNA transposons, also called “jumping genes,” are DNA sequences which typically move around the genome via a cut-and-paste mechanism (Muñoz-López and García-Pérez 2010). Retrotransposons are a kind of transposon which uses a copy-and-paste mechanism through an RNA intermediate to propagate within a genome (Finnegan 2012). As these mobile elements move within a genome, they generate a variety of mutations.

This motivates two questions:

1. How should we quantify the “distance” between two genomes when some mutations (e.g. mobile element insertions) represent single biological events rather than many independent changes?
2. Can such a distance measure detect biologically meaningful signals in real genomic data?

Classical unit-cost edit distance (Levenshtein 1966, Wagner and Fischer 1974) treats edited bases separately: a 400-base insertion costs 400 times as much as a single substitution, regardless of whether that insertion is a mobile element that inserted in a single biological event. Existing approaches address complementary parts of this problem: affine gap penalties discount multi-base indels but fail to adequately distinguish a known mobile-element sequence as a cohesive edit. Mobile element detection tools identify insertions but do not produce pairwise distances, and many alignment-free distance measures summarize sequence-wide similarity without assigning a specified motif its own block cost. This paper addresses these questions by specifying eligible motif operations and introducing a modified edit distance with a parameter m∈[0,1] controlling how strongly block events are discounted.

The suffix-based block-cost construction and an initial modified-distance definition were presented in H.K.S.’s University Honors capstone (Stoltz 2025). The present article substantially extends that work through explicit motif modes, a dynamic-programming recurrence and complexity analysis, an implementation, and empirical evaluations on mycobacteriophage genomes and the human PV92 locus.

### Contributions

- We provide an operational model for user-specified insertion-only and insertion–deletion motif blocks.
- We define a modified Levenshtein distance (mlev) with tunable block-event discounting and provide a dynamic-programming recurrence.
- We evaluate the method on 67 mycobacteriophage genomes (2211 pairs), confirming theoretical predictions and characterizing bidirectional score patterns for mycobacterium phage mobile elements 1 and 2 (MPME1 and MPME2).
- We demonstrate a second biological application at the human PV92 locus, with direction, related-motif, and reverse-complement controls.

#### 1.1 Related work

Affine gap penalties (Gotoh 1982) discount multi-base indels but treat all gaps equally regardless of sequence content. Modern exact aligners, including the wavefront algorithm, accelerate gap-affine alignment but retain a sequence-agnostic gap model (Marco-Sola *et al.* 2021). Block edit distances (Lopresti and Tomkins 1997; Ganczorz *et al.* 2018) allow substring operations but some variants are NP-hard in general, and they treat blocks as arbitrary substrings rather than biologically specified sequences.

Alignment-free methods compare sequences without a residue-by-residue alignment, using summaries such as *k*-mer profiles (Zielezinski *et al.* 2019), MinHash sketches (Ondov *et al.* 2016), or Lempel–Ziv relative information (Otu and Sayood 2003). Several established approaches can score insertions and deletions in sequence comparison, but the approaches above do not directly address the problem of discounting specific, known, mobile element sequences.

Mobile element detection tools [MELT (Gardner *et al.* 2017), RetroSeq (Keane *et al.* 2013), and xTea (Chu *et al.* 2021)] identify insertions from sequencing reads but do not produce pairwise distances. The mlev framework has a narrower, complementary role: it operates on assembled sequences and assigns discounted costs *only* to exact matching suffixes (end segments) of supplied element sequences, leaving non-motif gaps at full cost.

## 2 Methods

At a high level, MosaicLev takes two assembled DNA sequences, one or more user-supplied candidate mobile-element sequences, a scoring mode for each supplied sequence, and a discount parameter *m*. In insertion-only mode, matching element blocks can receive a reduced insertion cost; in insertion–deletion mode, both insertion and deletion can receive that reduced cost. It returns a sequence-comparison score. In the formal description below, *motif* means a supplied element sequence, *suffix* means an end segment of that sequence, and *block* means an exact matching suffix treated as one eligible block operation.

### 2.1 Motif model

We represent each genome as a string over the DNA alphabet Σ={A,T,C,G} and call each user-supplied candidate element sequence a *motif*. We use two scoring modes. In insertion–deletion mode, an eligible exact suffix of the motif can be inserted or deleted at reduced cost, reflecting a cut-and-paste model (Wicker *et al.* 2007, Muñoz-López and García-Pérez 2010).

In insertion-only mode, only insertion is discounted; deleting the same sequence still costs one edit per base, reflecting a copy-and-paste model in which the source copy remains (Wicker *et al.* 2007, Finnegan 2012).

This modeled biological asymmetry, where classical DNA transposons relocate while retrotransposons duplicate, directly motivates the cost structure in our directed score: transposon sequences receive discounted costs for both insertion and deletion, while retrotransposon sequences receive discounted insertion but full per-base deletion cost.

#### 2.1.1 Truncated insertions

Some L1 insertions lack part of their 5’ end because the reverse transcription process can terminate prematurely (Szak *et al.* 2002, Wicker *et al.* 2007). We therefore allow any *suffix* of the supplied motif to be inserted instead of the full string: any contiguous segment ending at the motif’s final base. For example, the suffixes of ATG are ATG, TG, and G. If $R=R_{1}\cdots R_{\left|R\right|}$, these suffixes are $R_{k}\cdots R_{\left|R\right|}$ for 1≤k≤|R|.

#### 2.1.2 User-specified motif modes

Motifs $R,T\in \Sigma^{+}$ are user-specified (forward orientation only). Membership in $M_{T}$ permits discounted insertion and deletion, while membership in $M_{R}$ permits discounted insertion only. This is a scoring assumption, not a molecular mechanism inferred from sequence. Here, $\Sigma^{+}$ denotes the set of nonempty DNA strings.

### 2.2 Modified Levenshtein distance

The classical Levenshtein distance function lev(a,b) (Levenshtein 1966, Wagner and Fischer 1974) is exactly defined as the minimum number of single-character insertions, deletions, or substitutions required to get from string *a* to string *b*. However, this unmodified Levenshtein function assigns base-by-base cost to retrotransposon copy–paste and DNA-transposon cut–paste operations. We therefore allow insertions or deletions of eligible mobile-element strings to cost between a single edit and the corresponding series of single-character operations.

So, it makes sense to assign a cost which scales somewhere between 1 and the block length. We introduce the parameter m∈[0,1], which controls the cost of eligible block events, and define the cost of a block edit of length *L* by


c(L)=(1−m)(L−1)+1.

In particular, c(1)=1. At m=0, our cost is c(L)=L, which is the same as ordinary Levenshtein. At m=1, our cost becomes c(L)=1, the same cost as a single point mutation. Researchers interested in intermediate weightings can interpolate linearly between the m=0 (standard Levenshtein) and m=1 values. This interpolation changes only the discount assigned to an eligible motif block. It does not estimate the probability of transposition or the fraction of biological change caused by an element.

#### 2.2.1 Setup (motif suffixes and costs)

Let *R* denote a forward-oriented motif assigned to insertion-only mode and *T* a forward-oriented motif assigned to insertion–deletion mode:


$$R=(R_{1})\cdots (R_{\left|R\right|}),\;T=(T_{1})\cdots (T_{\left|T\right|})$$

and a parameter m∈[0,1] controlling the discount.

We then use the following “suffix sets:”


$$M_{T}=\{(T_{k})\cdots (T_{\left|T\right|})|1\leq k\leq |T|\}$$

$$M_{R}=\{(R_{k})\cdots (R_{\left|R\right|})|1\leq k\leq |R|\}.$$

These sets contain every suffix ending at the final base of *R* or *T*.

#### 2.2.2 Allowed primitive edits

Given words over Σ={A,T,C,G}, we allow:

1. Single-base insertion, deletion, substitution (cost: 1).
2. *Block insertion* (transposons and retrotransposons): insert any block $M\in M_{T}\cup M_{R}$ (cost: c(|M|)).
3. *Block deletion (transposon only)*: delete any block $M\in M_{T}$ (cost c(|M|)).

We write cost(e) for the cost of a primitive edit *e*: single-base edits have cost 1, and any block insertion/deletion of a block *M* has cost c(|M|).

*Note: there is no discounted deletion for* $M_{R}$  *(insertion-only motif blocks). Biologically, retrotransposons use a copy–paste mechanism, so the source copy remains in the genome. Retrotransposon deletions are charged per base.*

#### 2.2.3 Definition

For $a,b\in \Sigma^{*}$, where $\Sigma^{*}$ is the set of all finite DNA strings, including the empty string ε, let E(a,b) be the set of all finite edit sequences (paths) that transform *a* into *b* using the primitive edits above. The modified distance is


$$\mathrm{mlev}(a,b)=\underset{\gamma \in E(a,b)}{\min}\sum_{e\in \gamma }\text{cost}(e).$$

Because insertion-only mode treats insertion and deletion differently, the score can change when sequence order is reversed: mlev(a,b) measures transformation from *a* to *b*, whereas mlev(b,a) measures the reverse transformation. We call each a directed score. For the phage pairwise matrices, we average the two directions, ${\mathrm{mlev}}_{\text{sym}}(a,b)=[\mathrm{mlev}(a,b)+\mathrm{mlev}(b,a)]/2$. For PV92, we report both directed scores and their average.

### 2.3 Dynamic-programming formulation of the modified Levenshtein

Write $a=a_{1}\cdots a_{p}$ and $b=b_{1}\cdots b_{q}$.

Let *D[i, j]* be the optimal cost to transform *a[*1..*i]* to *b[*1..*j]*, 0≤i≤p, 0≤j≤q, with D[0,0]=0.

The recurrence below selects the least costly available operation. Its first three interior terms represent a single-base deletion, insertion, and match or substitution. The final two are the MosaicLev additions: they delete or insert an exact matching suffix of a supplied element at the block cost. The indicator $1[a_{i}\neq b_{j}]$ equals 0 when the two bases match and 1 when they differ.


$$\begin{array}{c} D[i,0]=\min\{D[i-1,0]+1,\;\underset{\begin{array}{c} M\in M_{T},\;|M|\leq i\\ a[i-|M|+1..i]=M \end{array}}{\min}\;(D[i-|M|,0]+c(|M|))\},\\ D[0,j]=\min\{D[0,j-1]+1,\;\underset{\begin{array}{c} M\in M_{T}\cup M_{R},\;|M|\leq j\\ b[j-|M|+1..j]=M \end{array}}{\min}\;(D[0,j-|M|]+c(|M|))\},\\ D[i,j]=\min\left\{\begin{array}{c} D[i-1,j]+1,\\ D[i,j-1]+1,\\ D[i-1,j-1]+1[a_{i}\neq b_{j}],\\ \underset{\begin{array}{c} M\in M_{T},\;|M|\leq i\\ a[i-|M|+1..i]=M \end{array}}{\min}\;(D[i-|M|,j]+c(|M|)),\\ \underset{\begin{array}{c} M\in M_{T}\cup M_{R},\;|M|\leq j\\ b[j-|M|+1..j]=M \end{array}}{\min}(D[i,j-|M|]+c(|M|)) \end{array}\right\}. \end{array}$$

The value mlev(a, b) is D[p, q]. For complexity accounting, let A be the total number of eligible motif-suffix matches ending at positions in source sequence a, and let B be the corresponding total for target sequence b. With precomputed suffix matches, the core dynamic programming (DP) uses time *O*(*pq + qA + pB*) and full-matrix memory *O*(*pq*). For fixed motif sets, A=O(p) and B=O(q), so this bound is O(pq), matching the asymptotic time of classical edit-distance dynamic programming (Wagner and Fischer 1974). The implementation is available at https://doi.org/10.5281/zenodo.18452982 and was checked by deterministic regression examples and parameter sweeps. By construction, mlev(a,b)≤lev(a,b) for all *a*, *b*, with equality at m=0. All analyses here restrict *m* to this interval.

#### 2.3.1 Worked example

Let T=TTAA be a transposon motif, R=ATG a retrotransposon motif, and m=0.5. Then, c(3)=1+(0.5)(2)=2 and c(4)=1+(0.5)(3)=2.5. Consider:


$$\begin{array}{cc} \mathrm{mlev}(\varepsilon ,\mathrm{ATG}) & =c(3)=2<\mathrm{lev}(\varepsilon ,\mathrm{ATG})=3,\\ \mathrm{mlev}(\mathrm{TTAA},\varepsilon ) & =c(4)=2.5<\mathrm{lev}(\mathrm{TTAA},\varepsilon )=4,\\ \mathrm{mlev}(\mathrm{GC},\mathrm{ATGGC}) & =c(3)=2<\mathrm{lev}(\mathrm{GC},\mathrm{ATGGC})=3. \end{array}$$

In the third case, the optimal edit sequence inserts the retrotransposon suffix ATG at cost c(3)=2, rather than three single-character insertions at cost 3. At m=1, all three block operations would cost exactly 1 (a whole block acts like a single edit).

### 2.4 Biological datasets

We evaluated mlev on mycobacteriophage genomes containing two well-characterized mobile genetic elements. This provides exact motif references and negative controls rather than independent ground truth.

Mycobacteriophages are viruses that infect mycobacteria (Hatfull 2010). Cluster G phages (Pope *et al.* 2015) are closely related phages with an average genome size of ∼ 42 kb. Some contain a mobile element called MPME1 (mycobacterium phage mobile element 1, 439 bp), while others were originally described as lacking it (Sampson *et al.* 2009). A related element MPME2 (440 bp) also exists, sharing ∼78% sequence identity with MPME1 (Sampson *et al.* 2009). The originally described element-free Angel genome is ∼41 441 bp, while the MPME1-containing BPs genome is ∼41 901 bp (a difference of 460 bp). For computational analysis, we extracted a 434 bp analysis motif from the 439-bp MPME1 in the BPs genome at coordinates 39870–40303 (1-based and inclusive), beginning at the published left inverted-repeat boundary and omitting the final five bases of the right inverted repeat (see Data Availability for coordinates). Below, exact sequence searches replace genome length as the direct motif-content check. All MPME1 computations and direct-search labels used this same 434-bp motif.

We analyzed the 67 sequenced Cluster G1 mycobacteriophages downloaded from PhagesDB (Russell and Hatfull 2017) on 14 January 2026 (Supplementary Data). We focused on G1 because it contains the well-characterized MPME1 and MPME2 elements. This yields $\left(\begin{array}{c} 67\\ 2 \end{array}\right)=2211$ pairwise comparisons over the archived dataset. Based on genome size, seven phages with short genomes (∼ 41 077–41 456bp) were used as the reference group, while 60 phages formed the longer-genome group (∼ 41 650–44 145 bp). Genome length alone was not treated as proof of element identity (Table 1).

**Table 1:** Cluster G1 mycobacteriophage genome sizes and operational analysis groups.^a^

Phage	Size (bp)	Operational group
**Angel**	41 441	Reference
**CLED96**	41 456	Reference
**Cherrybomb426**	41 456	Reference
**GoldenAsh**	41 441	Reference
**Schiebel**	41 441	Reference
**Taheera**	41 077	Reference
**Terror**	41 077	Reference
**60 phages with longer genomes (representative examples)**		
**BPs**	41 901	Long
**Hope**	41 901	Long
**Zombie**	41 901	Long
**BruceB**	41 901	Long
**Halo**	42 289	Long^b^

a Seven short genomes define the reference group, and 60 longer genomes define the comparison group. This grouping does not independently label mobile element content.

b Halo contains the extracted MPME2 sequence.

This yields three comparison categories: 21 reference–reference pairs (negative controls), 420 reference–long-genome pairs, and 1770 long-genome–long-genome pairs. MPME1 was extracted from the BPs genome (GenBank EU568876) and MPME2 from Halo (GenBank DQ398042), both originally characterized by Sampson *et al*. (2009). We use each motif in a separate suffix set $M_{T}$ to test target specificity.

#### 2.4.1 Phage score groups and direct-search labels

Applying MosaicLev separately with MPME1 and MPME2 divided the 60 non-reference phages at the 20% decision boundaries into three groups: a 31-genome MPME1 group (high MPME1, low MPME2), an 11-genome MPME2-score group (low MPME1, high MPME2), and an 18-genome double-low group. These groups are based on mlev discount patterns, not independent biological labels. Additionally, we searched every genome in both orientations and defined an MPME1-positive label before receiver operating characteristic (ROC) calculation as an exact suffix match covering at least 90% of the 434-bp MPME1 motif. This corresponds to at least 391 consecutive bases ending at the motif’s final base. This rule labeled 31 of the 60 non-reference phages positive and 29 negative. Per-genome scores were means over comparisons with the seven references. The ROC used the mean MosaicLev percentage cost reduction, negative mean ordinary Levenshtein distance, raw unnormalized mean Needleman–Wunsch global-alignment score, and negative mean 21-mer Jaccard distance. The affine-gap calculation used match =1, mismatch =−1, a gap-open penalty of 10, and a gap-extension penalty of 1; the 21-mer calculation used sets of distinct 21-mers from the stored forward-oriented sequences. Thus, larger values predicted an exact-suffix-positive genome for all four scores.

### 2.5 Implementation

For full genome comparison (**∼** 41–44 kb sequences), the core O(pq) dynamic-programming grid requires **∼** 1.7–$1.9\times 10^{9}$ cell evaluations and 13–15 GB of matrix storage per pair. We implemented an optimized algorithm using Numba JIT compilation (Lam *et al.* 2015) that precomputes suffix positions before the DP phase, achieving ∼50× speedup over pure Python (∼34 seconds versus ∼30 minutes per mlev computation). Pairwise computations were parallelized across the NYU Greene HPC cluster (16 GB memory per job) using SLURM job arrays. Each of the 2211 pairs was computed independently with both MPME1 and MPME2 motifs, sweeping the discount parameter *m* over {0.0,0.1,0.2,…,1.0}. Total computation required **∼** 500 core-hours. ROC curves and areas under the curve were computed by the supplied deterministic analysis script. All reported phage matrices use the average of the two comparison directions.

### 2.6 PV92 human-locus application

For a second biological application, we used the nested human PV92 Alu polymorphism described by Comas *et al*. (2001) and the deposited 788-bp BAS101 sequence (GenBank AF302689.1). The observed nested state contains an outer Ya5 Alu and an inner Yb8 insertion, allowing us to apply the previously demonstrated techniques. We formed an AF302689.1-matched pre-Yb8 state of 442 bp by deleting coordinates 220–565 and a matched empty state of 129 bp by additionally deleting reconstructed-Ya5 coordinates 86–398. These are controlled reconstructions, not observed ancestors. All AF302689.1 coordinates are 1-based and inclusive.

The exact net inserted blocks were the 346-bp nested Yb8 block (335-bp Yb8 body plus the downstream 11-bp target-site near-copy) and the 313-bp outer Ya5 block (306-bp Ya5 body plus the downstream exact 7-bp target-site copy). Both were supplied in the insertion-only $M_{R}$ mode. For each event, we cycled through m=0,0.1,…,1 in the forward and reverse directions and tested the related Alu motif and the reverse complement as controls. We report forward and reverse scores, their arithmetic mean, and the forward percentage reduction $100[\mathrm{lev}-{\mathrm{mlev}}_{\text{forward}}]/\mathrm{lev}$, where forward transforms the reconstructed state without the insertion into the state containing it. All three sequence states are shorter than 800 bp.

## 3 Results

We quantify the effect of mlev using the *discount percentage*: for each pair (a,b), discount =100[lev(a,b)−s(a,b)]/lev(a,b), where s=mlev for a directed analysis and $s={\mathrm{mlev}}_{\text{sym}}$ for the phage pair matrices. If lev(a,b)=0, we define the discount as 0. This represents the mathematical reduction in assigned edit cost relative to ordinary Levenshtein distance, not the fraction of biological change caused by a mobile element. Table 2 shows representative pairwise results at m=0.5 and m=1.0.

**Table 2:** Representative pairwise distances.^a^

Pair	Category	lev	${\mathrm{mlev}}_{m=0.5}$	${\mathrm{mlev}}_{m=1}$	**Reduction at** m=1
**Angel versus Schiebel**	Reference	1	1.0	1.0	0.0%
**Angel versus GoldenAsh**	Reference	67	67.0	67.0	0.0%
**CLED96 versus Terror**	Reference	6061	5951.5	5842.0	3.6%
**Angel versus BPs**	Ref.–exact MPME1	618	400.5	183.0	70.4%
**Angel versus Hope**	Ref.–exact MPME1	619	401.5	184.0	70.3%
**BPs versus Schiebel**	Ref.–exact MPME1	617	399.5	182.0	70.5%
**Angel versus Halo**	Ref.–exact MPME2	1582	1523.0	1464.0	7.5%
**BPs versus BruceB**	Long–long	50	50.0	50.0	0.0%
**BPs versus Hope**	Long–long	581	457.0	23.0	96.0%
**BPs versus Phish**	Long–long	822	459.5	26.0	96.8%
**Frosty24 versus Hope**	Long–long	702	618.5	343.0	51.1%

a “Reference” denotes two members of the seven-genome reference group. “Ref.–exact MPME1/2” denotes a reference compared with a genome containing the complete exact motif. “Long–long” denotes two members of the longer-genome group. Discount is computed at m=1.0. The Halo pair demonstrates that mlev assigns little MPME1-specific reduction to a genome containing the related MPME2 element (see text).

Table 3 summarizes the mean discount by threshold-defined score group for cross-type comparisons (reference phages versus non-reference phages, 420 pairs).

**Table 3:** Score summary for separate mlev analyzes using MPME1 (434 bp motif) and MPME2 (440 bp) as target motifs.^a^

		**Mean cost reduction (** m = 1.0 **)**	
Comparison type	Pairs	MPME1 motif	MPME2 motif
**Reference versus MPME1-score group**	217	53.1% ± 33.7%	3.5% ± 1.2%
**Reference versus MPME2-score group**	77	9.0% ± 3.7%	51.7% ± 34.7%
**Reference versus low/low-score group**	126	9.6% ± 7.0%	2.9% ± 1.1%

a Cross-type comparisons between the seven original reference phages and the threshold-defined groups reveal clear score separation: the MPME1-score group (31 phages) shows high discount with MPME1 motif but low with MPME2, while the MPME2-score group (11 phages) shows the reverse pattern. The low/low-score row (126 pairs = 7×18) compares the seven references against 18 phages below 20% on both motif axes. Population standard deviations are reported for the pairwise values. These descriptive groups require direct sequence evidence before biological assignment.

**Figure 1:**
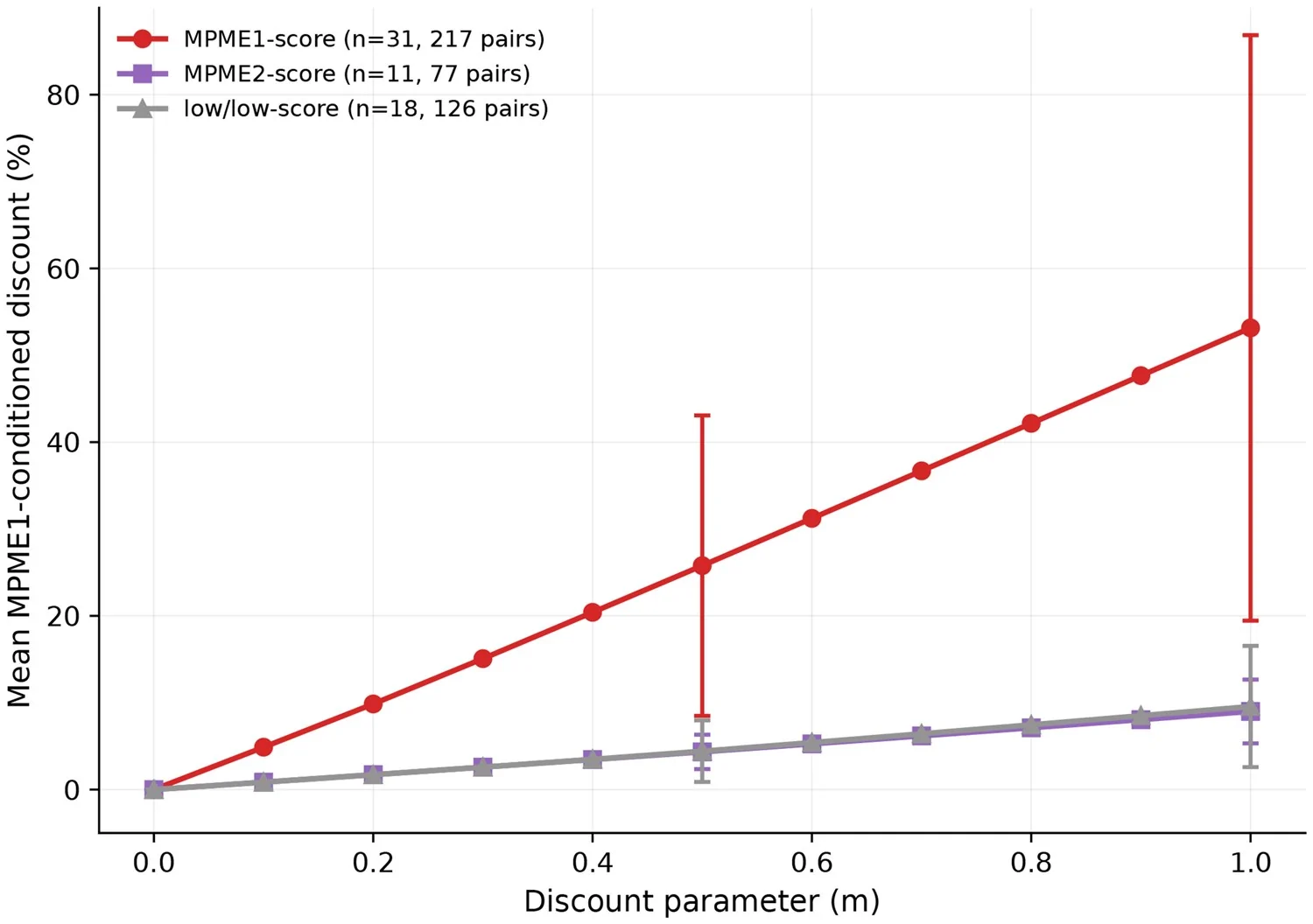
Mean discount percentage as a function of the discount parameter m for cross-type comparisons (reference phages versus the three score groups). All three categories show monotone increases: at m=0 (standard Levenshtein), discount is zero, and at m=1 (full discounting), the MPME1-score group reaches ∼53% mean discount. The large error bars for this group reflect the bimodal distribution shown in Figure 3. Error bars are population standard deviations over the 217, 77, and 126 reference- versus-group pair values. The MPME2-score and low/low-score groups follow similar trajectories (∼9%−10% at m=1), showing low MPME1-conditioned reduction and that MPME1 analysis alone cannot distinguish them.

The standard Levenshtein assigns distances of 618 to Angel–BPs, 1582 to Angel–Halo, and 581 to BPs–Hope, without identifying how much of each total is compatible with the supplied motif. mlev lowers these costs by 70%, 7%, and 96%, respectively, when the indicated element is supplied. Direct searches find exact MPME1 in BPs and Hope and exact MPME2 in Halo. Figure 1 shows that the discount increases monotonically with *m*, with all categories converging to zero at m=0 (standard Levenshtein). The parameter *m* provides tunability: higher values discount eligible mobile element events more heavily relative to point mutations. We emphasize m=1 for group summaries unless otherwise noted, which treats a full block insertion as a single edit operation and provides the clearest biological interpretation.

**Figure 2:**
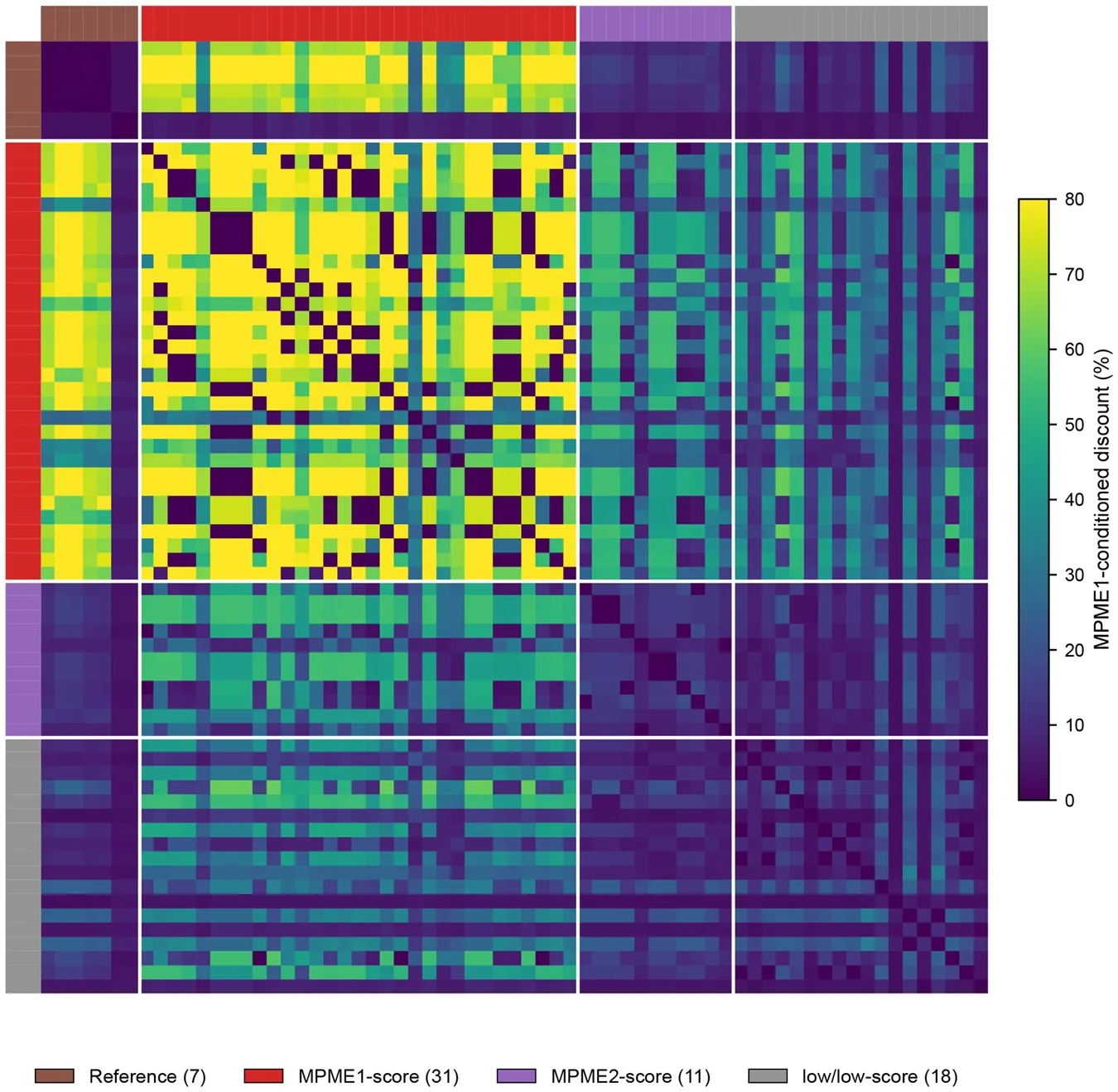
Heatmap of pairwise discount percentages at m=1.0 for all 67 Cluster G1 phages (MPME1 motif). Phages are grouped by analysis group: reference (brown, seven phages), MPME1-score (red, 31 phages), MPME2-score (violet, 11 phages), and low/low-score (gray, 18 phages). White lines delineate category boundaries. The block structure reveals clear score differences: the Reference × MPME1-score block has the highest mean discount (53.1%), while Reference × MPME2-score and low/low-score blocks have lower means (9.0% and 9.6%). The MPME1-score × MPME1-score block (center) displays a striking checkerboard pattern of heterogeneous reductions, and the diagonal is uniformly dark (self-comparisons yield zero discount).

Figure 2 provides an overview of all 2211 pairwise comparisons. Rows and columns list the same 67 genomes in reference, MPME1-score, MPME2-score, and low/low-score order. Each cell gives the discount calculated from the average of the two comparison directions for that pair at m=1: dark cells are near zero and yellow cells are high. White lines separate groups, and the dark diagonal contains self-comparisons. The block structure immediately reveals the score differences: the reference–MPME1-score block is brightest on average, whereas the reference–MPME2-score and reference–low/low-score blocks are darker. Most striking is the checkerboard pattern within the MPME1-score × MPME1-score block, which records heterogeneous pairwise score reductions.

### 3.1 Phage score patterns and concordance

#### 3.1.1 Method validation

Negative controls (21 reference–reference pairs where direct searches found no near-complete MPME1) show minimal discount: mean 1.9% at m=1, likely reflecting chance suffix matches with the 434-suffix motif set. This serves as the noise floor. Cross-type pairs with canonical MPME1 show the expected discount: at m=1, a 434 bp block edit costs c(434)=1 instead of 434 single-character operations, yielding an expected absolute discount of 433 edits. For a typical cross-type pair with lev ≈ 618, this corresponds to 433/618 ≈ 70%. Observed values for canonical exact-match examples (e.g. Angel versus BPs at 70.4%) closely match this prediction.

**Figure 3:**
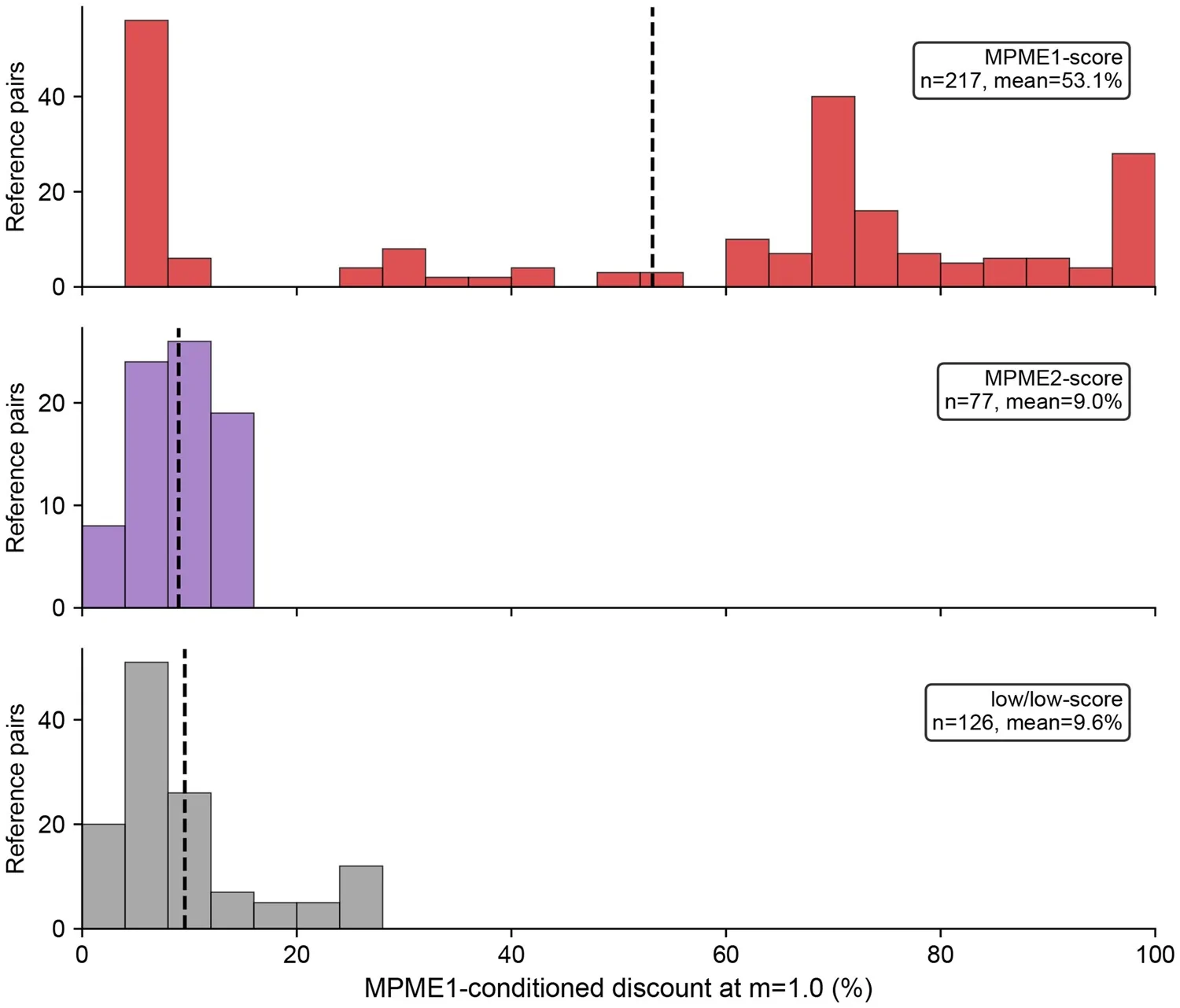
Distribution of discounts in cross-type comparisons (reference phage versus score-group member) using the MPME1 motif. The MPME1-score group (red, n=217) shows a striking bimodal distribution: one mode lies at 65–100% discount while a second lies at 0%–20%. This bimodality explains the high standard deviation (±33.7%) and why the mean (53.1%) does not represent a typical value. The MPME2-score group (violet, n=77) and the low/low-score group (gray, n=126) both show distributions clustered at low values (generally 5%–15%). These groups are indistinguishable by MPME1 analysis alone, motivating the two-motif score plot in Figure 4.

Figure 3 reveals that the MPME1 score-group distribution is *bimodal*, not normal: one mode lies at 65%–100% while a second lies at 0%–20%. The bimodal structure is quantitatively evident: 62 pairs (29%) fall in the low mode (<25% discount) while 135 pairs (62%) fall in the high mode (>50% discount), with only 20 pairs (9%) in between. This bimodality explains the category mean (53.1%) and high standard deviation (±33.7%): the mean does not represent a typical value but rather falls between two distinct subpopulations. Importantly, the MPME2-score and low/low-score groups show similar low-score distributions (means 9.0% and 9.6%).

#### 3.1.2 MPME1/MPME2 score patterns

**Figure 4:**
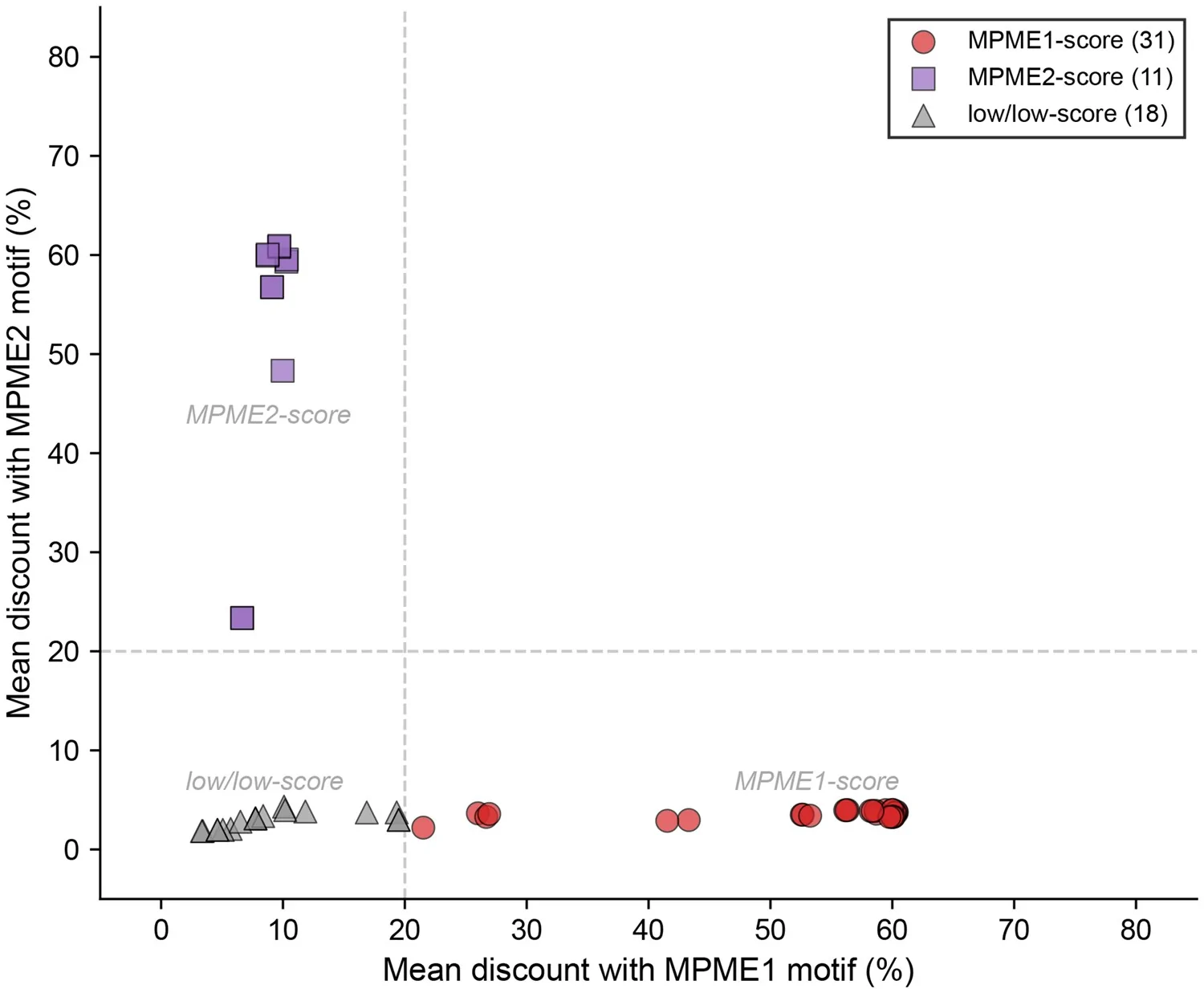
MPME1-versus-MPME2 score plot. Each point represents one of 60 non-reference phages: coordinates are mean discounts averaged across comparisons with all seven reference phages. X axis: discount with MPME1 motif. Y axis: discount with MPME2 motif. The three threshold-defined groups occupy distinct quadrants by definition: the MPME1-score group (red circles, n=31) clusters in the lower-right (high MPME1 discount, low MPME2), the MPME2-score group (violet squares, n=11) in the upper-left, and the low/low-score group (gray triangles, n=18) in the lower-left corner. Dashed lines at 20% indicate the descriptive decision boundaries.

To examine discrimination of MPME1 from MPME2, we performed a second analysis using the MPME2 element (440 bp, extracted from Halo genome DQ398042) as the target motif. Figure 4 shows each non-reference phage plotted by its mean discount (averaged across all seven reference phages) with the MPME1 motif (*x*-axis) versus the MPME2 motif (*y*-axis). The 20% decision boundaries partition the three score groups into distinct quadrants by definition. The MPME1-score group (red circles) clusters in the lower-right quadrant with high MPME1 discount (20%–65%) but uniformly low MPME2 discount (∼3%–5%). The MPME2-score group (violet squares) shows the inverse pattern in the upper-left quadrant: low MPME1 discount (∼7%–12%) but high MPME2 discount (23%–62%). Halo lies near 23% despite containing a 100% exact 440-bp MPME2 match, illustrating that the percentage also depends on all other pairwise differences.

The 18 phages in the lower-left quadrant (gray triangles) show low discount with *both* motifs (<20% on both axes), forming a cluster near the origin. These phages, Aroostook, Barkley26, Chance64, Frosty24, Gideon, Grizzly, Hotshotbaby7, Jolene, Jonghyun, Liefie, LouisV14, Paito, Periodt, Rabbs, Rattrick, Renaissance, Ruriko, and Sneeze, show low motif-specific discount but cannot be assigned as element-free from this result alone.

#### 3.1.3 Sequence-level checks

When the element is at the *same* position in both genomes (e.g. BPs versus BruceB), no block edit is needed and we observe zero discount. Direct exact searches place the complete MPME1 motif at the same coordinate in that pair and at different coordinates in BPs and Phish, whose discount is 96.8%. These comparisons are compatible with positional differences. Direct sequence comparison identifies one mismatch in Frosty24 at 1-based motif position 369, which left a 65-bp exact suffix and an intermediate score. The scalar mlev value is sensitive to the interrupted match.

#### 3.1.4 Exact suffix concordance

We performed direct sequence searches for MPME1 and MPME2 motifs in each genome. The fixed 90% exact-MPME1-suffix rule labeled 31 of the 60 non-reference phages positive and 29 negative. The 20% MPME1-score threshold agreed for 54/60 phages (90.0%): LouisV14, Periodt, and Rattrick were exact-suffix positive but below the score threshold, whereas Camri, Phloodle, and Phreak were exact-suffix negative but above it. All seven reference phages show <50% match to both motifs.

MPME2 targeting produced a reciprocal, motif-selective pattern. The 11-genome MPME2-score group averaged 51.7% discount with the MPME2 motif but only 9.0% with MPME1. Conversely, the 31-genome MPME1-score group averaged 53.1% with MPME1 and 3.5% with MPME2, while the low/low-score group remained low with both motifs (9.6% and 2.9%). So, changing only the supplied motif clearly distinguished the score patterns of two related elements sharing **∼** 78% sequence identity. MPME2 targeting also separated the MPME2-score and low/low-score groups, which had nearly identical scores under MPME1 targeting. All 11 members of the MPME2-score group contained a near-complete (at least 90%) MPME2 motif match.

#### 3.1.5 Comparator performance

**Figure 5:**
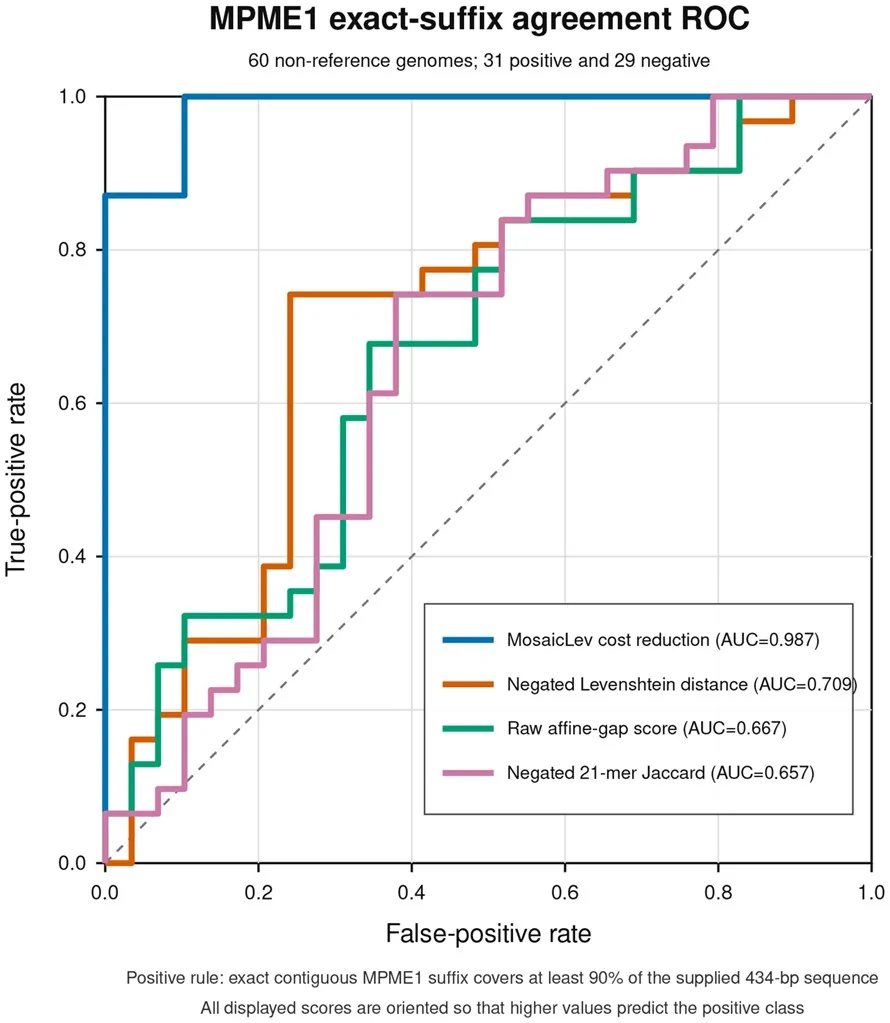
ROC curves for concordance with the 90% exact-MPME1-suffix rule using per-genome mean scores (n=60 non-reference phages: 31 positive and 29 negative). Each phage’s score is its mean value averaged across all seven reference comparisons, so the ROC uses 60 genome-level targets rather than 420 pairwise rows. Shared references and relatedness mean these targets are not statistically independent. The mlev discount (solid blue, AUC = 0.99) is compared with standard Levenshtein distance (solid orange, AUC = 0.71), affine gap alignment (solid green, AUC = 0.67), and 21-mer Jaccard distance (solid purple, AUC = 0.66). Levenshtein and Jaccard distances were negated for the ROC calculation; the raw affine-gap score was not normalized. The diagonal dotted line represents random classification (AUC = 0.50).

To evaluate concordance with the fixed 90% exact-suffix rule, we used ROC analysis (Fawcett 2006). An area under the receiver operating characteristic curve (AUC) of 1 indicates perfect separation, whereas 0.5 indicates chance-level performance. Because the exact-suffix labels and the MosaicLev discount both use the supplied MPME1 sequence, this analysis measures concordance with that rule rather than independent biological validation. To avoid treating the 420 pairwise rows as separate targets, we computed per-genome mean discounts (averaged across all seven reference comparisons), yielding 60 genome-level observations. These observations share the same seven references and phylogenetic relatedness and are not statistically independent. We compared the mlev discount with three standard pairwise comparators: standard Levenshtein distance, affine-gap alignment score, and 21-mer Jaccard distance. Figure 5 shows the resulting ROC curves. The mlev discount achieves AUC = 0.99, compared with Levenshtein (AUC = 0.71), affine gap (AUC = 0.67), and 21-mer Jaccard distance (AUC = 0.66). At the 20% discount threshold used throughout this paper, mlev achieves 89.7% specificity and 90.3% sensitivity (28 true positives, 3 false negatives, 26 true negatives, and 3 false positives).

### 3.2 PV92 locus-scale results

**Figure 6:**
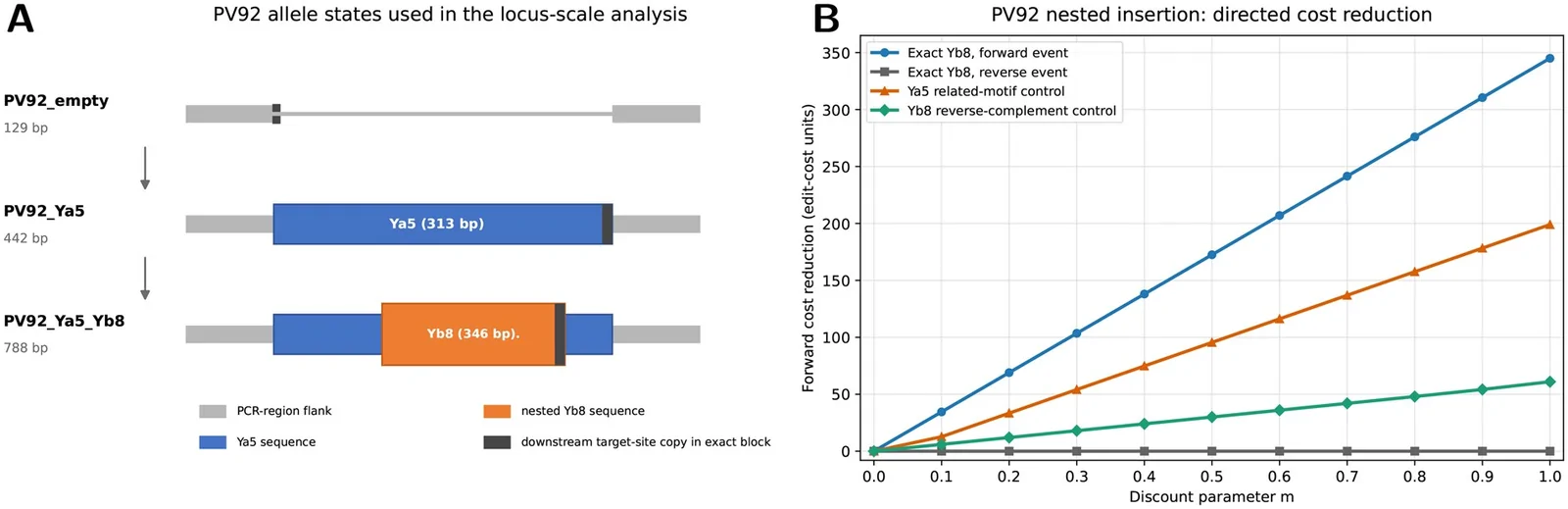
PV92 human-locus application. (a) The observed deposited Ya5 + Yb8 sequence and the two matched reconstructed states: colored blocks show the exact net inserted Ya5 and Yb8 sequences, including downstream target-site copies. (b) Directed cost reduction across m=0,0.1,…,1 for the nested Yb8 event, its reverse direction, the related Ya5 motif, and the Yb8 reverse complement. The exact forward Yb8 curve rises most steeply, while the reverse direction remains at zero reduction.

The PV92 application asks whether the same implementation assigns its strongest directed reduction to known nested Alu insertion blocks. Figure 6 shows the observed and reconstructed states and the nested Yb8 parameter sweep, and Table 4 gives both events and all controls. At m=0, every run equals ordinary Levenshtein distance. At m=1, the exact 346-bp Yb8 forward event falls from 346 to 1 (99.71% reduction), whereas the reverse event remains 346. The exact 313-bp Ya5 forward event similarly falls from 313 to 1 (99.68%), whereas the reverse remains 313. This is the expected insertion-only directionality.

**Table 4:** Directed PV92 insertion-only results.^a^

Event	Motif/direction	Lev	**MLev** m=0.5	**MLev** m=1	**Reduction at** m=1
**Nested Yb8**	Exact Yb8, forward	346	173.5	1	99.71%
**Nested Yb8**	Exact Yb8, reverse	346	346.0	346	0.00%
**Nested Yb8**	Ya5 related-motif control	346	250.5	147	57.51%
**Nested Yb8**	Yb8 reverse-complement control	346	316.0	285	17.63%
**Outer Ya5**	Exact Ya5, forward	313	157.0	1	99.68%
**Outer Ya5**	Exact Ya5, reverse	313	313.0	313	0.00%
**Outer Ya5**	Yb8 related-motif control	313	302.5	291	7.03%
**Outer Ya5**	Ya5 reverse-complement control	313	300.0	287	8.31%

a “Lev” is ordinary Levenshtein distance. Reduction is the assigned-cost reduction relative to Lev. The substantial Ya5 control response for the nested event is retained because related Alu sequences share exact suffixes.

The controls expose the intended boundary of exact suffix matching. For the nested event at m=1, the related Ya5 motif still reduces the score by 57.51%, although the complete Ya5 block is absent from the target, because related Alu sequences share exact suffixes. The Yb8 reverse complement reduces it by 17.63%. Outer-event controls are lower (7.03% and 8.31%). Averaging the forward and reverse scores gives 173.5 for exact Yb8 and 157 for exact Ya5 at m=1, because the undiscounted reverse direction is averaged with the forward score. The inner 11-bp target context is a near-copy with one mismatch, and the matched Ya5 state retains an unresolved nucleotide whose timing relative to Yb8 insertion is unknown. The result therefore validates a controlled locus-scale scoring behavior, not ancestry, mechanism, population classification, or chromosome-scale performance.

## 4 Discussion

### 4.1 Comparison with existing methods

Table 5 summarizes the primary outputs of mlev relative to existing approaches. Standard edit and alignment methods can represent insertions, but they fail to adequately distinguish a known mobile-element sequence as a cohesive edit. Alignment-free methods, including Lempel–Ziv and MinHash-based measures, efficiently summarize overall sequence similarity but do not condition the score on one supplied motif. Mobile element detection tools (MELT, RetroSeq, xTea) solve a different problem: identifying mobile-element insertions and producing calls or genotypes from read or platform-specific evidence in a reference workflow, not computing pairwise distances between assembled sequences. Block edit distances allow substring operations but are broader formulations and are not necessarily motif specific. The mlev framework instead provides a directed pairwise score from assembled sequences plus explicit motif input. In the exact-suffix concordance analysis (Fig. 5), its AUC is 0.99 compared with 0.71 for standard Levenshtein, 0.67 for affine gap, and 0.66 for 21-mer distance. This is a task-specific comparison, not evidence that MosaicLev replaces alignment, alignment-free, or insertion-calling tools.

**Table 5:** Comparison of method roles.^a^

Method	Primary output	Supplied-motif block cost	Typical input
**Levenshtein**	Edit distance	No	Assembled sequence
**Affine gap (Gotoh 1982)**	Alignment/score	No	Sequence
**Alignment-free LZ/MinHash (Otu and Sayood 2003; Ondov *et al.* 2016)**	Overall sequence distance	No	Sequence/sketch
**Block edit (Lopresti and Tomkins 1997)**	Block-aware distance	Not necessarily	Sequence
**MELT/RetroSeq/xTea (Keane *et al.* 2013; Gardner *et al.* 2017) (Chu *et al.* 2021)**	Insertion calls	No	Reads plus reference
**MosaicLev**	Directed comparison score	Yes	Sequence plus motif

a MosaicLev supplies a pairwise comparison score that incorporates a supplied element sequence, while the other method classes solve complementary alignment, aggregate-comparison, block-edit, or insertion-calling tasks.

### 4.2 Limitations

The current implementation has several limitations. First, the exact implementation is practical for complete genomes in the tens-of-kilobases range and for bounded loci within larger genomes. Its quadratic time and memory requirements preclude end-to-end human-genome comparisons, so the PV92 analysis is a locus-scale demonstration rather than whole-genome validation. Larger genomes will require windowed or approximate methods. Second, mlev detects only exact suffix matches to the motif set: a mismatch can shorten the eligible suffix, and highly divergent element copies may escape detection entirely. Third, the suffix-based approach cannot distinguish between a true mobile element insertion and an unrelated sequence that happens to match the motif suffixes by chance, though our negative controls suggest this contributed little on average (1.9% mean discount in reference–reference pairs). Fourth, the implementation returns a scalar cost without a traceback, so it cannot by itself localize an event, distinguish competing mechanisms, or support a single-SNP interpretation of Frosty24. Finally, insertion-only mode makes the score direction-dependent: mlev(a,b) and mlev(b,a) can differ. The phage matrices average the two comparison directions, whereas PV92 reports both directions.

### 4.3 Biological implications

The bimodal distribution of discounts among the MPME1-score group (Fig. 3) reveals unexpected score heterogeneity. Candidate explanations include different insertion positions, sequence variants, truncated copies, and unrelated background differences, but the scalar score does not choose among them. This variation in the scores obtained with the supplied MPME1 sequence is not separated from other differences by standard edit distance, which treats all 31 members of this score group using the same basewise costs. The low/low scores of 18 phages despite their expanded genomes (>41.5 kb) raise intriguing questions: do these phages harbor a third, uncharacterized mobile element? Or do their size differences reflect accumulated small indels unrelated to mobile element activity? The present analysis leaves that question open and specifies the local-alignment, annotation, and breakpoint evidence needed to answer it.

At PV92, the exact forward Alu blocks received the largest reductions, reverse insertion-only comparisons received none, and the related-Ya5 control showed that shared suffixes can produce partial reductions. MosaicLev is, therefore, best used to test a specified candidate event, not to discover or classify unknown events on its own.

### 4.4 Future directions

Natural extensions include incorporating multiple motif variants in the suffix sets to track several mobile element families simultaneously, investigating whether approximate methods [suffix-tree matching (Ukkonen 1993) or seed-and-extend heuristics (Altschul *et al.* 1990)] can be adapted to scale to larger genomes, and using a similar version of our formalism, adapted for transposition-like viral events, to ask how closely the model can reconstruct viral lineages. The framework could also be extended to weight different motif families differently, allowing simultaneous tracking of elements with different biological impacts. For human pangenomes, an initial route would be to compare anchored locus windows containing a prespecified candidate insertion before attempting chromosome-scale computation. This requires validation across diverse haplotypes and is not demonstrated here.

## 5 Conclusion

We specified insertion–deletion and insertion-only motif modes and introduced a modified Levenshtein dissimilarity mlev with tunable block-event discounting. Evaluation on 67 mycobacteriophage genomes confirmed the cost-function prediction: representative exact-match cross-type pairs with canonical MPME1 show the expected **∼** 70% discount, while negative controls remain near zero. Separate analyses using MPME1 and MPME2 as target motifs partition the genomes into descriptive score groups. Changing the supplied motif produced complementary score patterns, and direct searches found near-complete MPME2 matches in all 11 genomes in the MPME2-score group. The MPME1 score threshold agrees with the fixed exact-suffix rule for 90.0% of non-reference genomes. At the human PV92 locus, known exact Ya5 and Yb8 forward insertion blocks receive more than 99.6% directed cost reduction at m=1, whereas their reverse events receive none. By recognizing known mobile elements as cohesive edits, MosaicLev captures genomic changes that are obscured by standard sequence comparisons.

## Supplementary Material

vbag261_Supplementary_Data

## Data Availability

The mlev implementation is available at https://doi.org/10.5281/zenodo.18452982 (source code: https://github.com/kuhlman-lab-ucr/Genetic-Mosaicism). Mycobacteriophage genome sequences were obtained from PhagesDB (https://phagesdb.org). The MPME1 motif sequence (434 bp) was extracted from the BPs genome (GenBank accession EU568876, coordinates 39870–40303, 1-based and inclusive), and the MPME2 motif sequence (440 bp) was extracted from the Halo genome (GenBank accession DQ398042, coordinates 38875–39314, 1-based and inclusive). The published 439-bp MPME1 element extends through BPs coordinate 40308. The MPME1_Position and MPME2_Position columns in the Supplementary Data direct-search table contain zero-based start indices. Analysis materials are provided in the Supplementary Data. The observed PV92 nested allele is GenBank AF302689.1. Exact reconstruction coordinates, derived sequence states, motifs, controls, result tables, and reproduction scripts are included in the Supplementary Data. The ROC labels, per-genome scores, and plotting script are also included.
